# Time Window Determination for Inference of Time-Varying Dynamics: Application to Cardiorespiratory Interaction

**DOI:** 10.3389/fphys.2020.00341

**Published:** 2020-04-28

**Authors:** Dushko Lukarski, Margarita Ginovska, Hristina Spasevska, Tomislav Stankovski

**Affiliations:** ^1^Faculty of Medicine, Ss. Cyril and Methodius University, Skopje, Macedonia; ^2^University Clinic for Radiotherapy and Oncology, Skopje, Macedonia; ^3^Faculty of Electrical Engineering and Information Technologies, Ss. Cyril and Methodius University, Skopje, Macedonia; ^4^Department of Physics, Lancaster University, Lancaster, United Kingdom

**Keywords:** time-series analysis, dynamical systems, dynamical Bayesian inference, coupled oscillators, coupling functions

## Abstract

Interacting dynamical systems abound in nature, with examples ranging from biology and population dynamics, through physics and chemistry, to communications and climate. Often their states, parameters and functions are time-varying, because such systems interact with other systems and the environment, exchanging information and matter. A common problem when analysing time-series data from dynamical systems is how to determine the length of the time window for the analysis. When one needs to follow the time-variability of the dynamics, or the dynamical parameters and functions, the time window needs to be resolved first. We tackled this problem by introducing a method for adaptive determination of the time window for interacting oscillators, as modeled and scaled for the cardiorespiratory interaction. By investigating a system of coupled phase oscillators and utilizing the Dynamical Bayesian Inference method, we propose a procedure to determine the time window and the propagation parameter of the covariance matrix. The optimal values are determined so that the inferred parameters follow the dynamics of the actual ones and at the same time the error of the inference represented by the covariance matrix is minimal. The effectiveness of the methodology is presented on a system of coupled limit-cycle oscillators and on the cardiorespiratory interaction. Three cases of cardiorespiratory interaction were considered—measurement with spontaneous free breathing, one with periodic sine breathing and one with a-periodic time-varying breathing. The results showed that the cardiorespiratory coupling strength and similarity of form of coupling functions have greater values for slower breathing, and this variability follows continuously the change of the breathing frequency. The method can be applied effectively to other time-varying oscillatory interactions and carries important implications for analysis of general dynamical systems.

## 1. Introduction

Dynamical systems are widespread in nature, with examples including biological, chemical, climatological and social systems. Often they interact with other systems and the environment, exchanging information and matter (Winfree, 1980; Haken, 1983; Kuramoto, 1984; Pikovsky et al., 2001; Strogatz, 2001). This makes their states, parameters and functions time-varying (Kloeden and Rasmussen, 2011; Stankovski, 2013; Suprunenko et al., 2013; Lehnertz et al., 2014).

Biological dynamical systems form an important group of such systems. They are the central focus to medicine and biomedicine. Different physiological systems reflect the function of human bodily organs and processes, directly linked to various states and diseases (Peskin, 1981; Levy et al., 2006). Understanding and being able to detect certain physiological characteristics of such systems and functions is thus of great importance and relevance to science with direct implications for the human well-being.

Such biological systems are usually not isolated, but interact between each other (Bashan et al., 2012). The cardiorespiratory interaction, as central mechanism of the cardiovascular system, has been studied extensively in relation to different states and diseases (Schäfer et al., 1998; Stefanovska et al., 2000; Stankovski et al., 2012; Iatsenko et al., 2013; Kralemann et al., 2013a; Schulz et al., 2018; Grote et al., 2019). The cardiac and the respiration signals can be acquired by non-invasive measurements, making the investigations of cardiorespiratory interaction easily accessible. Both systems have periodic oscillatory dynamics, which makes them also very convenient for modeling in terms of their phase dynamics (Rosenblum et al., 2002; Stankovski et al., 2012; Kralemann et al., 2013a; Ticcinelli et al., 2017). Similarly to the other open biological systems, the dynamics of the cardiorespiratory system can also be time-varying, including a situation where the frequency, the coupling strength or the coupling function are varying in time—which adds a challenging complexity when analysing such data.

Different aspects of the cardiorespiratory interaction have been studied, including phase synchronization, coupling strength/directionality and the coupling functions (Rosenblum et al., 2002; Paluš and Stefanovska, 2003; Voss et al., 2008; Stankovski et al., 2012; Kralemann et al., 2013a; Hagos et al., 2019). The latter describe the functional mechanism of how the interactions occur and develop (Stankovski et al., 2017). As such, the coupling functions have attracted much attention recently, with many publications describing novel aspects of interaction mechanisms of the cardiorespiratory and other interactions across different scientific fields (Kiss et al., 2007; Ranganathan et al., 2014; Stankovski et al., 2014b; Ashwin et al., 2019; Moon and Wettlaufer, 2019; Rosenblum et al., 2019). The main focus of the current paper will be also on coupling functions and how to infer optimally their time-variability.

Even though physiological dynamical systems, including the all-important cardiorespiratory interaction, are of great value and importance, when analysing their data, inevitable, one faces a *problem of how to determine the length of the time window*. Namely, when analysing the time-series data one needs to be able to follow the time-variability of the dynamics, i.e., the dynamical parameters and functions, but in order to do so, one needs to determine first the length of the time window. Then the data are usually analyzed through consecutive time windows, i.e., data portions of the time-series. Here, the length of the window will determine the time-resolution of the resulting parameters and functions. The main requirement for the window length is usually a tradeoff between (i) long enough time window to have the required amount of data for the methods to work correctly and (ii) short enough time window to get as good as possible time-resolution of the resulting parameters and functions. These conflicting requirements, (i) and (ii), make the choice for the window length very difficult and ambiguous, hence, usually, the time window length is a free parameter and it is chosen based on the subjective experience and intuition of the expert analyst.

In this paper, we developed a procedure for determination of the time window based on data analyses, as opposed to the previous practice of arbitrary choice. We extend a method for Dynamical Bayesian Inference of time-varying dynamics in the presence of noise, to utilize the inferred covariance matrix in order to determine the best choice of the time window. The choice is based on the inferred results as a tradeoff between low parameter error and low noise strength error. The method is tested and demonstrated on numerical phase and limit-cycle oscillators and on time-varying cardiorespiratory interactions.

## 2. Methods and Modeling Results

### 2.1. Dynamical Bayesian Inference

In the context of the method of interest, the dynamical inference refers to a model inference that will describe the solution of a system of differential equations via time series analysis. When two oscillators interact sufficiently weakly, their motion is effectively approximated with their phase dynamics (Kuramoto, 1984; Nakao et al., 2014). If we describe the system phase as a generic monotonic change of the variables, the dynamical process can be presented as:

(1) $$\begin{array}{l} \overset{•}{\varphi_{i}}=\omega_{i}+q_{i}\left(\varphi_{i},\varphi_{j}\right)+\xi_{i}, \end{array}$$

where φ_*i*_ is the phase of the i-th oscillator, ω_*i*_ is its phase velocity, *q*_*i*_ is the coupling function between the two oscillators, and ξ_*i*_ is the noise. It is assumed that the noise is white Gaussian ξ_*i*_(*t*)ξ_*j*_(τ) = δ(*t* − τ)*E*_*ij*_, where the symmetric matrix *E*_*ij*_ incorporates the information about the correlation between the noises of the different oscillators.

The periodic behavior of the system indicates that the coupling function can be represented by a Fourier decomposition:

(2) $$\begin{array}{l} q_{i}\left(\varphi_{i},\varphi_{j}\right)=\overset{\infty }{\underset{k=1}{\sum }}\overset{\infty }{\underset{s=1}{\sum }}c_{i;k,s}e^{i2\pi k\varphi_{i}}e^{i2\pi s\varphi_{j}} \end{array}$$

Usually, the dynamics will be well-described by a finite number K of Fourier terms, hence Equation (1) can be written as:

(3) $$\begin{array}{l} \overset{•}{\varphi_{i}}=\overset{K}{\underset{k=-K}{\sum }}{c_{k}}^{i}\Phi_{i,k}\left(\varphi_{i},\varphi_{j}\right)+\xi_{i}\left(t\right), \end{array}$$

where $i=\left\{1,2\right\},\Phi_{1,0}=\Phi_{2,0}=1,{c_{0}}^{i}=\omega_{i}$ and the rest Φ_*i,k*_ and ${c_{k}}^{i}$ are the K most important Fourier components (in this work we used *K* = 2). If a white Gaussian noise is assumed 〈ξ_*i*_(*t*)ξ_*j*_(τ)〉 = δ(*t* − τ)*E*_*ij*_, the task is than reduced to inference of the unknown parameters of the model:

(4) $$\begin{array}{l} M=\left\{{c_{k}}^{i},E_{ij}\right\}. \end{array}$$

For a given time series of observed phases χ = {φ_*i,n*_≡φ_*i*_(*t*_*n*_)}, (*t*_*n*_ = *nh, i* = 1, 2), the Bayesian statistics allows us to determine the posterior density, using the prior density *p*_*prior*_(*M*) as well as a likelihood function *l*(χ|*M*):

(5) $$\begin{array}{l} p_{\chi }\left(M|\chi \right)=\frac{l\left(\chi |M\right)p_{prior}\left(M\right)}{\int l\left(\chi |M\right)p_{prior}\left(M\right)dM}. \end{array}$$

In the Dynamical Bayesian Inference (Smelyanskiy et al., 2005; Duggento et al., 2012; Stankovski et al., 2012, 2014a) one makes certain initial assumptions about the parameters of the model that describes the observed time series. Then, the Bayesian theorem is successively applied in a recursive stepwise manner and in each following step of the inference, the inferred model parameters are getting closer to their real value. With each step of the inference, one obtains the value of the concentration matrix Ξ (which is the inverse of the covariance matrix Ξ = Σ^−1^).

#### 2.1.1. The Challenge of the Time Window and the Propagation Parameter

When using the aforementioned method, the time series of the phases of the oscillators are acquired by measurements followed by signal processing. The time series can be considered as time sequences of blocks of samples. Each block incorporates the samples in a certain time interval, hence the duration of the block determines the time window *t*_*w*_. The Bayesian inference is performed for each block and values for the parameters of the model and the couplings of the oscillators are obtained. The output values of the previous block are used as input values for the inference of the current block.

The method comprises a dynamical inference, so it needs to follow the time evolution of the set of parameters *c* and at the same time to enable separation of the dynamical effects from the noise. To achieve such separation, in the propagation sequence of the method, the input covariance matrix for the following block $\Sigma_{prior}^{\left(n+1\right)}$ is not taken as simply equal to the output covariance matrix of the current block $\Sigma_{post}^{n}$, but it is modified by the diffusion matrix Σ_*diff*_. The diffusion matrix is defined by the normal diffusion of each of the parameters. Hence, the input covariance matrix for the following block is a convolution of the two current normal distributions $\Sigma_{prior}^{\left(n+1\right)}=\Sigma_{post}^{n}+\Sigma_{diff}$ (Duggento et al., 2012; Stankovski et al., 2012). The covariance matrix Σ_*diff*_ describes which part of the dynamical field defined by the oscillators is changed and the intensity of those changes. The elements of this matrix are given by (_Σ_*diff*_)(*i, j*)_ = ρ_*ij*_σ_*i*_σ_*j*_, where σ_*i*_ is the standard deviation of the diffusion of the parameter *c*_*i*_, after time window *t*_*w*_ from the previous to the next block of samples, and ρ_(*i,j*)_ gives the correlation between the changes of the parameters *c*_*i*_ and *c*_*j*_. A special case is investigated, when there is no correlation between the parameters, i.e., ρ_(*i,j*)_ = 0, for *i* ≠ *j* and each standard deviation σ_*i*_ is a known fraction of the corresponding parameter *c*_*i*_: σ_*i*_ = *p*_*w*_*c*_*i*_, where *p*_*w*_, called the propagation parameter, is a constant parameter. The index *w* in *p*_*w*_ emphasizes that the propagation parameter is determined for a time window of length *t*_*w*_. In this way the propagation parameter defines how much variability should the method search for and infer. Being an input in the covariance matrix Σ_*diff*_ it expresses our belief about which part of the dynamics has changed, and the extent of that change. This is a tradeoff between inferring correctly the time-varying parameters and not inferring too much random noise perturbations. In the earlier works, this propagation parameter *p*_*w*_ was a free parameter chosen arbitrarily.

In the method of Dynamical Bayesian Inference (Duggento et al., 2012; Stankovski et al., 2012) the time window and the propagation parameter are free parameters and they are arbitrarily selected. The purpose of this research is to propose a method to determine the values of these two parameter in order to optimize the inference of the parameters and the noise.

As an indicator of quality of the inference the covariance matrix Σ is used. By definition, this is a matrix whose element in the (*i, j*) position is the covariance between the i-th and j-th element of a multidimensional random vector. The elements on the main diagonal of the covariance matrix are the variances of the variables, i.e., the covariance of each element with itself. Since the square of the variance is the standard deviation, by minimizing the sum of squares of the elements of the covariance matrix we are minimizing the standard deviations of the inferred model parameters. Therefore, we use the sum of squares of all the elements of the covariance matrix $Q_{\Sigma }=Sum_{i,j}\left({\Sigma_{i,j}}^{2}\right)$, called quadrature covariance matrix, as an indicator of deviations of the inferred parameters from the real intrinsic parameters.

### 2.2. Determination of the Time Window

In order to developed and present the procedure for determination of the time window we investigate first two coupled phase oscillators in presence of noise:

(6) $$\begin{array}{ll} \overset{•}{\varphi_{1}} & =\omega_{1}\left(t\right)+a_{1}sin\left(\varphi_{1}\right)+a_{3}\left(t\right)sin\left(\varphi_{2}\right)+\sqrt{E_{11}}\xi_{11}\left(t\right)\\ \overset{•}{\varphi_{2}} & =\omega_{2}+a_{2}sin\left(\varphi_{1}\right)+a_{4}sin\left(\varphi_{2}\right)+\sqrt{E_{22}}\xi_{22}\left(t\right). \end{array}$$

Here, ω_1_ and ω_2_ are parameters for the angular frequency of the corresponding oscillators, *a*_1_ and *a*_4_ are the parameters of their own dynamics, and *a*_2_ and *a*_3_ are the coupling parameters for the direct influence from the other oscillator. Two of the parameters are varied periodically in time, the frequency ω_1_(*t*) and the coupling parameter *a*_3_(*t*). Uncorrelated Gaussian white noises are used. In this way the true values of the parameters of the oscillatory systems are known in advance.

From these oscillatory systems we generate numerical signals which we then introduce as input data for the Dynamical Bayesian Inference. As a result we obtain the inferred values of the parameters and the noise, as well as the quadrature matrix *Q*_Σ_ for each block of the inference. Apart from *Q*_Σ_, we evaluate the error difference between the inferred parameters *c*_*i*_ and their true values $\tilde{c_{i}}$: $\Delta c_{i}=c_{i}-\tilde{c_{i}}$, and the same was done with the noise strengths $\Delta E_{i}=E_{i}-\tilde{E_{i}}$. We investigate the dependance of *Q*_Σ_, Δ*c*_*i*_ and Δ*E*_*i*_ on the time window *t*_*w*_, for different values of the propagation parameter *p*_*w*_.

For the system of two coupled phase oscillators (Equation 6) we simulated multiple time series of 2,000 s each, with sampling step *h* = 0.01, corresponding to a 10 ms step. These time series are the input data for the Dynamical Bayesian Inference. In the study the parameters *a*_1_, *a*_2_, and *a*_4_ are constant: *a*_1_ = 0.8, *a*_2_ = 0, and *a*_4_ = 0.6. The frequency ω_2_ was varied in the interval from 0.785 to 31.4. The time-varying parameters are given by:

(7) $$\begin{array}{ll} \omega_{1} & =\omega_{1,const}-0.5sin2\pi f_{1}t\\ a_{3}=a_{3,const}-0.3sin\left(2\pi f_{3}t+\pi /2\right), \end{array}$$

where *a*_3,*const*_ was either 0.8 or 1.3, ω_1,*const*_ was varied in the interval 0.785–62.8, and the oscillator frequencies *f*_1_ and *f*_2_ were changed in the interval 0.001–0.02. For the noise (*E*_11_, *E*_22_) values in the interval (0.01, 10) were taken. For these values we investigated the dependence of *Q*_Σ_, Δ*c*_*i*_ and Δ*E*_*i*_ on the time window *t*_*w*_ and the propagation parameter *p*_*w*_.

**Figure 1:**
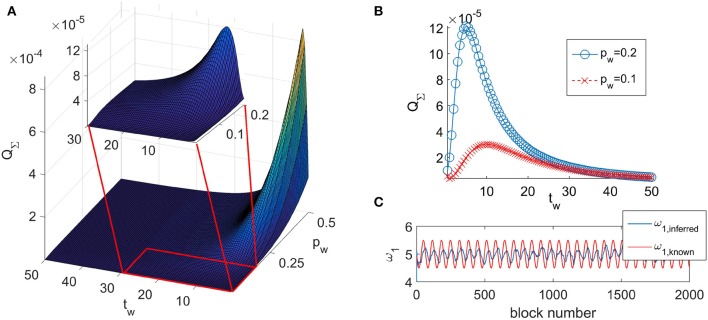
**(A)** Typical look of the dependance of *Q*_Σ_ on the time window *t*_*w*_ and the propagation parameter *p*_*w*_ for coupled phase oscillators (Equation 6). **(B)** *Q*_Σ_ as a function of the time window *t*_*w*_ for two different values of the propagation parameter *p*_*w*_. **(C)** The inferred value of a parameter ω_1,*inferred*_ and the known value of the same parameter ω_1,*known*_.

The typical look of the dependance of *Q*_Σ_ on the time window *t*_*w*_ and the propagation parameter *p*_*w*_ is given in Figure 1A. The function of the quadrature matrix *Q*_Σ_ on the time window *t*_*w*_ shows a maximum that depends on the value of the propagation parameter *p*_*w*_. We have determined that the maximum is obtained for the value of the time window *t*_*w,max*_ = 1/*p*_*w*_. As the relationship shows, with decreasing value of *p*_*w*_, the maximum is shifted to greater values of *t*_*w*_ (as shown on Figure 1B).

The performed analysis showed that for all combinations of *t*_*w*_ and *p*_*w*_ that place the inference on the left of the maximum (*t*_*w*_< *t*_*w,max*_) of the corresponding curve *Q*_Σ_(*t*_*w*_), the inference does not follow the time change of the parameters—shown on Figure 1C. It appears that such combinations of *t*_*w*_ and *p*_*w*_ do not allow the inference to reach the amplitude of change of the time-varying parameter. We will call this behavior as the delayed-inference regime.

For *t*_*w*_ > *t*_*w,max*_, the value of *Q*_Σ_ steadily decreases (as shown in Figure 1) and the deviations of the inferred parameters from their true values also decrease. However, values for the time window that are too large also prevent appropriate inference of the time changes of the parameters simply because there are too few blocks for their representation.

Based on these results we conclude that the time window should have a value as high as possible, in order for *Q*_Σ_ to be as low as possible, but at the same time a value that is still low enough to be able to accurately represent the dynamic of the parameter that is changing with the highest frequency. Therefore, in the analysis, we performed an initial estimation of the time change of the parameters of the model by using a small arbitrary value for the time window and an initial value of the propagation parameter *p*_*w*_ = 0.2. We use small time window in order for inferred parameters to be able to describe the fast changes of their true values. Then we performed a fast Fourier transform on the initial estimation of the parameters from which we determined the highest frequency of the time-varying change of the parameters. We denote this frequency as *f*_*max*_ and the corresponding period as *T*_*min*_ = 1/*f*_*max*_. From our analysis of the time-varying ability we concluded that the minimal number of blocks needed to accurately describe this fastest changing parameter is eight blocks, i.e., the time window should be taken as $t_{w,opt}=\frac{T_{min}}{8}=\frac{1}{8f_{max}}$. That will give a resolution of eight points to describe the fastest oscillating inferred parameter. For all the other parameters there will be more points describing their oscillations.

### 2.3. Determination of the Propagation Parameter

From the numerical analysis we determined that the inferred covariance matrix *Q*_Σ_ increases with the increase of the propagation parameter *p*_*w*_ up to saturation for very big values of *p*_*w*_ (*p*_*w*_ > 7 in our simulations). Hence, in order to get the best possible inference, we should use the smallest possible propagation parameter. However, as we have shown in Figure 1, for small propagation parameter, smaller than *p*_*w,min*_ = 1/*t*_*w,max*_ the inference does not follow the time change of the parameters and is in the delayed-inference regime.

To determine the optimal value of the propagation parameter we have investigated the difference between the inferred parameters and their known value. We have evaluated this difference in two different ways.

One was to look at the graphs like the one shown in Figure 1C for different values of the time window *t*_*w*_ and by evaluating the difference between the inferred parameter and its known value to determine the minimal value for *t*_*w*_ for which the inferred parameter starts to follow the change of the known parameter. This will be the *t*_*w*_ value when the Δ*c*_*i*_ stops manifesting periodic changes in time.

The second way was to calculate the mean square error (MSE) between the time series of the inferred parameter and the time series of its known value (excluding the first two blocks of the inference). The mean square error was calculated for different values of the propagation parameter and a graph *MSE* = *f*(*p*_*w*_) was constructed for different *t*_*w*_ = *t*_*w,opt*_ values. These graphs showed a minimum that gives the *p*_*w*_ value for which the correspondence between the inferred and the known value of the parameters is the best.

**Figure 2:**
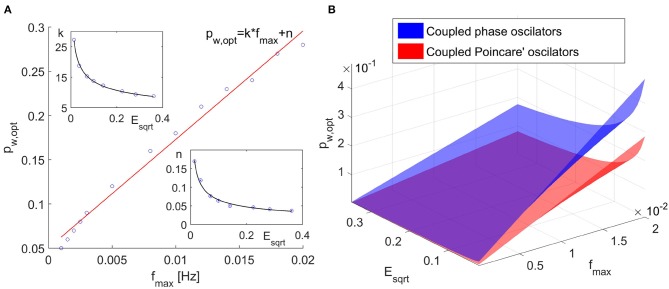
**(A)** Optimal propagation parameter *p*_*w,opt*_ as a function on the maximal frequency of the parameter change. **(B)** Optimal propagation parameter as a function of the maximal frequency of parameter change and the noise for coupled phase oscillators and coupled Poincaré oscillators.

We have performed this evaluation for different frequencies of change of the parameters of the model and for different noises. The time window values used in these simulations were the optimal values (*t*_*w,opt*_). From these analysis we have found that the optimal value for the propagation parameter depends both on the frequencies of the changes of the parameters (i.e., on the optimal time window) and on the noise. Further, we have found that the optimal value of the propagation parameter is approximately linearly dependent on the frequency of the fastest changing parameter *f*_*max*_ (Figure 2A). The slope and the intercept of the linear function were found to depend on the noise. This dependence can approximately be described by inverse power law (Figure 2A).

From the numerical analysis we have determined that we can relate the optimal propagation parameter, *p*_*w,opt*_, to the optimal time window, *t*_*w,opt*_. As a rule, the optimal propagation parameter needs to be greater than the reciprocal optimal time window *p*_*w,opt*_ > 1/*t*_*w,opt*_. Further more, in the interval of frequencies and noises that we investigated, which are of interest and corresponds to cardiorespiratory interactions, the propagation parameter in the Dynamical Bayesian Inference can be selected as follows. For slow dynamics, when the optimal time window is >40 s, one can use the value *p*_*w,opt*_ = 0.1 as optimal propagation parameter. For optimal time windows in the interval *t*_*w,opt*_ ∈ (10*s*, 40*s*), one can use the value *p*_*w,opt*_ = 0.2 as optimal propagation parameter. For fast dynamics, when the optimal time window is <10 s, the optimal propagation parameter should be calculated as *p*_*w,opt*_ = 2/*t*_*w,opt*_. We emphasize that these values can be used for cardiorespiratory interactions when the noise is not too small. With decreasing noise, one needs to take increasingly higher values for the optimal propagation parameter.

### 2.4. Algorithm for the Optimization of Time Window and Propagation Parameter Values

Based on the results obtained in sections 2.2 and 2.3 we propose the following algorithm for determining of the optimal time window *t*_*w,opt*_ and propagation parameter *p*_*w,opt*_.

Using a small arbitrary value for the time window and an initial value for the propagation parameter of *p*_*w*_ = 0.2 we perform an initial inference. The arbitrary value for the time window can be the smallest value at which the method gives an output. For values smaller than this arbitrary value of *t*_*w*_ the Bayesian inference will not work (the execution of the code will give a “Singular matrix error”, because the concentration matrix will be too small). In this way we will obtain the initial inferred parameters *c*_*ij*_ and noises *E*_*ij*_ that describe the model. This inference will have the best information on the parameter dynamics in terms of time-variation, but the parameter noise will be quite large. Then we perform a fast Fourier transform of the inferred parameters *c*_*ij*_. By observing both the dynamic of each of the parameters and their fast Fourier transform, we are able to determine what the highest frequency of change of the parameters is. We denote this frequency as *f*_*max*_. The corresponding period is *T*_*min*_ = 1/*f*_*max*_. By assuming the minimal number of blocks needed to accurately describe this fastest changing parameter, the time window should be taken as *t*_*w,opt*_ = *T*_*min*_/8 = 1/8*f*_*max*_. This will give a resolution of eight points to describe the fastest oscillating inferred parameter. For all the other parameters there will be more points describing their oscillations.

Based on the value of the optimal time window, for the case scaled around the frequencies in the cardiorespiratory range, when the noise is not too small, we can determine the optimal propagation parameter as:

(8) $$\begin{array}{l} p_{w,opt}=\{\begin{array}{ll} 0.1, & t_{w,opt}>40\\ 0.2, & t_{w,opt}\in [10,40]\\ \frac{2}{t_{w,opt}}, & t_{w,opt}<10. \end{array} \end{array}$$

With these values for *t*_*w,opt*_ and *p*_*w,opt*_ we perform a second, optimized inference. In this inference the covariance matrix will have smaller value, thus resulting in an improved inference.

### 2.5. Analysis of Coupled Limit-Cycle Oscillators

To test the proposed algorithm for determination of the time window and the propagation parameter, we investigate a system of two coupled limit-cycle oscillators – Poincaré oscillators subject to white noise:

(9) $$\begin{array}{l} {\dot{x}}_{1}=-\left(\sqrt{x_{1}^{2}+y_{1}^{2}}-1\right)x_{1}-\omega_{1}(t)y_{1}+\varepsilon_{1}(x_{2}-x_{1})+\xi_{1}(t)\\ {\dot{y}}_{1}=-\left(\sqrt{x_{1}^{2}+y_{1}^{2}}-1\right)y_{1}+\omega_{1}(t)x_{1}+\varepsilon_{1}(y_{2}-y_{1})+\xi_{2}(t)\\ {\dot{x}}_{2}=-\left(\sqrt{x_{2}^{2}+y_{2}^{2}}-1\right)x_{2}-\omega_{2}y_{2}+\varepsilon_{2}(t)(x_{1}-x_{2})+\xi_{3}(t)\\ {\dot{y}}_{2}=-\left(\sqrt{x_{2}^{2}+y_{2}^{2}}-1\right)y_{2}+\omega_{2}x_{2}+\varepsilon_{2}(t)(y_{1}-y_{2})+\xi_{4}(t), \end{array}$$

where periodic time-variability is introduced in the frequency of the first oscillator ω_1_(*t*) = 1−0.4*sin*(2π*f*_1_*t*) and in the coupling parameter from the first to the second oscillator ε_2_(*t*) = 0.2−0.1*sin*(2π*f*_2_*t*). The noises are again white and Gaussian, with no correlations between them and were changed in the interval *E*_*i*_ ∈ [0.005, 0.05], *i* = {1, 2, 3, 4}. The other parameters are ω_2_ = 4.91 and ε_1_ = 0.05. The frequency of the time-variability was changed in the interval *f*_*i*_ ∈ [0.0015, 0.02], *i* = {1, 2}.

**Figure 3:**
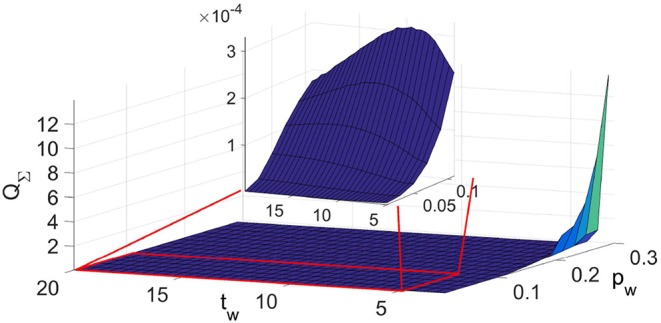
Typical look of the dependance of quadrature matrix *Q*_Σ_ on the time window *t*_*w*_ and the propagation parameter *p*_*w*_ for coupled Poincaré oscillators.

In Figure 3 we show the quadrature covariance matrix as a function of the time window and the propagation parameter.

As in the case of the coupled phase oscillators, here as well we see a maximum in the function of the quadrature covariance matrix *Q*_Σ_ on the time window *t*_*w*_ that depends on the value of the propagation parameter as *t*_*w,max*_ = 1/*p*_*w*_. Again, the performed analysis showed that for values of *t*_*w*_ smaller than the value for the maximum of the curve, *t*_*w,max*_, regardless of the value of the propagation parameter, the inference does not follow the time change of the parameters.

The results for the propagation parameter also showed increase in the inferred quadrature matrix *Q*_Σ_ with the increase of the propagation parameter *p*_*w*_ and by implementing the same analysis as in the case of coupled phase oscillators, we found that the optimal propagation parameter increases linearly with the increase of the maximal frequency change of the parameters. Again the slope and the intercept of the line *p*_*w,opt*_ = *k* * *f*_*max*_ + *n* showed decrease with increasing noise and the decrease can be approximated with inverse power law. As expected, we determined different values for the coefficients of the inverse power laws. However, these coefficients always yielded values for the propagation parameter *p*_*w,opt*_ smaller than the one for the coupled phase oscillators, as shown in Figure 2B, hence the determination of the propagation parameter according to the Equation (8) will give satisfactory results.

## 3. Application to Cardiorespiratory Interaction

It is well-appreciated that the cardiac and respiration dynamics are oscillating, while being part of the multi-system body they are not isolated, but they are open systems where their parameters and functions are time-varying (Glass, 2001; Stankovski et al., 2012; Kralemann et al., 2013b; Rosenblum et al., 2019). The oscillatory nature makes them suitable to be represented with the phase dynamics (Kuramoto, 1984; Nakao, 2016). These two aspect of the cardiorespiratory dynamics, the oscillatory phase dynamics and their time-variability, make the proposed method of dynamical Bayesian inference with adaptive time window very good fit for such analysis.

In order to demonstrate the potential of the method on experimental data, we analyzed cardiorespiratory measurements conducted on one male subject, age 35, non-smoker without cardiovascular health issues. The study was reviewed and approved by Ethical Committee, Faculty of Medicine, Saints Cyril and Methodius, Skopje, Macedonia and the participant provided written informed consent that the collected data might be used and published for research purposes. The respiration followed a predetermined pattern by following a visual and audio computer simulation in which a ball was moved along a sine line on a computer screen. The frequency of the movement of the ball, together with the sine line, was changing according to the law that we wanted the respiration to follow. When the ball was reaching the maximum and minimum of the sine line, a short sound beep was also generated. The measurements were performed using Biopac equipment with the subject in supine position. The respiration was measured by placing a respiratory transducer on the chest of the subject measuring the changes in the chest circumference, while the cardiac function was recorded by performing a three-lead ECG measurement.

Three different patterns of respiration were studied and compared: spontaneous free breathing, time-varying breathing following a sine wave and time-varying breathing following a-periodic signal. The average respiratory rates of the investigated respiratory patterns were 14.7 BrPM (Breaths per Minute) for the spontaneous free breathing, 15.5 BrPM for the respiration following a sine law and 17.0 BrPM for the breathing following the aperiodic signal. These average respiratory rates correspond to average respiratory frequencies of 0.245, 0.258, and 0.283 Hz, respectively. The corresponding average heart rates were found to be 68.3 BPM (Beats per Minute), 69.0 and 77.4 BPM for the spontaneous free breathing, periodic and aperiodic respiration, respectively.

**Figure 4:**
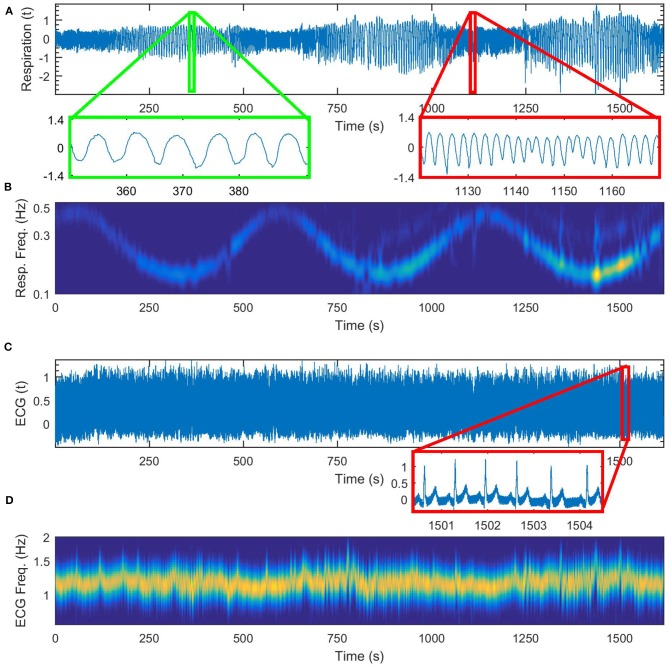
Cardiorespiratory measurements for a time-varying respiration following a sine law, **(A)** respiration signal, **(B)** wavelet transform of the respiration signal, **(C)** ECG signal, **(D)** wavelet transform of the ECG signal.

In Figure 4 we show first in detail the cardiorespiratory measurements for a time varying respiration following a simple sine law. The frequency of respiration is varied according to the law *f* = 0.3+0.2*sin*(2π*t*/560), [Hz]. The time-varying perturbed respiration signal is shown in Figure 4A and its wavelet transform is given in Figure 4B. On Figure 4C we give the corresponding ECG signal and on Figure 4D the wavelet transform of the cardiac signal.

**Figure 5:**
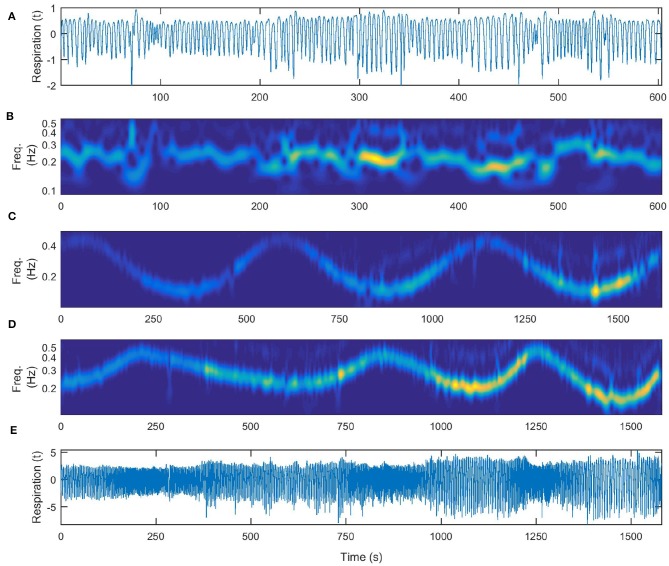
Time-varying nature of the respiration measurements. **(A)** Respiratory signal recorded during free breathing. **(B)** Wavelet transform of the free breathing respiration. **(C)** Wavelet transform of time-varying respiration following a simple sine law (for comparison the same as depicted in Figure 4B). **(D)** Wavelet transform of time-varying respiration following an a-periodic behavior, **(E)** the same recorded signal of time-varying respiration following an a-periodic behavior.

In Figure 5 we show the signals and their wavelet transforms for the three different patterns of respiration that the subject followed: (a) respiration signal recorded during free breathing, (b) time-frequency wavelet transform of the free breathing respiration, (c) wavelet transform of time varying respiration following a simple sine law (the same as depicted in Figure 4B for comparison), (d) wavelet transform of time varying respiration following an a-periodic behavior and the signal itself (e). The a-periodic signal was taken to be the z-component of a chaotic Lorenz system (Lorenz, 1963).

After the wavelet power inspection of the measurements we performed the phase extraction procedure. For robust phase extraction, the oscillating intervals were estimated by standard digital filtering procedures, including a FIR filter followed by a zero-phase filtering procedure (filtfilt) to ensure that no time or phase lags were introduced by the filtering. The boundary of the interval for the respiration signal was *r* = 0.145–0.6 Hz; and boundary of the interval for the heart activity from the ECG signal was *h* = 0.6–2 Hz (Kralemann et al., 2008; Shiogai et al., 2010; Stankovski et al., 2016). The phases of the filtered signals were estimated by use of the Hilbert transform, and the protophase-to-phase transformation Kralemann et al. (2008) was then applied to the resultant protophases to obtain invariant observable-independent phases.

In the case of free breathing, as can be seen in Figure 5B, there was no single frequency dominating the time variance of the parameters. Therefore, when we did the first inference of our algorithm, higher frequencies emerged in the variance of the parameters and in their Fast Fourier Transform. Since we wanted to include the higher frequencies in the consequent investigation we had to use smaller time windows, as our algorithm suggests (*t*_*w,opt*_ = 9*s*). This increased the covariance matrix, but at the same time faster changes were included in the inference and we were able to follow better the time evolution of the parameter change and of the coupling functions. In the case of time varying respiration according to the sine law, as is the case of Figure 4, the frequency of change of the respiration dominated the first inference. This led to higher optimal time window (*t*_*w,opt*_ = 62*s*) and to a second inference with reduced covariance matrix. In the case of time varying respiration according to a-periodic law, as is the case of Figures 5D,E, again the algorithm gave smaller values for the optimal time window (*t*_*w,opt*_ = 15.6*s*), that enabled inclusion of different frequencies of change of the parameters at the cost of increased covariance matrix.

Finally we present the application results of our method for the cardiorespiratory coupling. Once we have determined the optimal values for the time window and the propagation parameter we can proceed with the inference of the parameters of the model *c*_*i*_, from which we can calculate the coupling quantities and characteristics. We evaluated the coupling functions on a 2π × 2π grid using the relevant base functions, i.e., Fourier components scaled by their inferred coupling parameters. We calculated the coupling strength *CPL*_*i*_(*t*) as the Euclidian norm of the inferred parameters for a particular coupling. Importantly, we also calculated the index for similarity of coupling functions ρ(*t*) which quantifies the similarity of the forms of two coupling functions irrespectively of their coupling strength amplitudes. The similarity index is unique measure of coupling functions and it is calculated as correlation index between the vectors *c*_*i*_ of two coupling functions (Kralemann et al., 2013a; Ticcinelli et al., 2017). It is important to note that the coupling strength and the similarity index present two different dimensions of a coupling function (Stankovski, 2017). In our analysis we calculated the similarity index between the time-average coupling function and every coupling function calculated from each time window—in this way we got the time-variability of the form of the coupling function as compared to the average coupling function.

**Figure 6:**
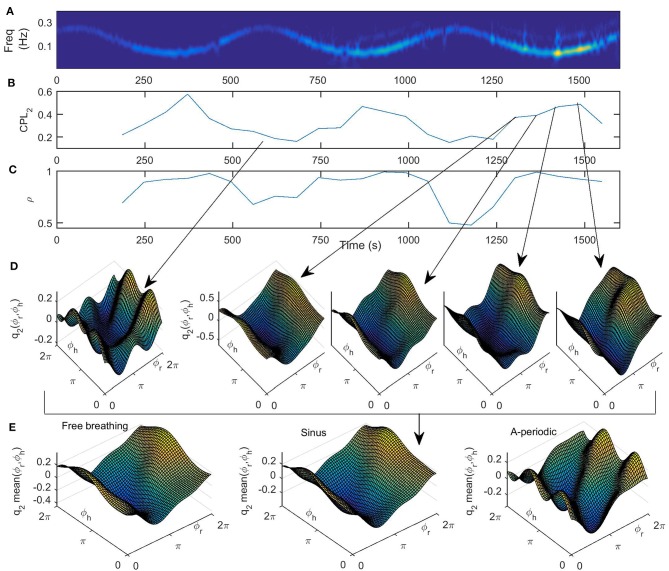
**(A)** Wavelet transform of time-varying respiration following a simple sine law, **(B)** the time variation of the coupling strength *CPL*_2_(*t*) from the first oscillator (the respiratory system) to the second one (cardiac system), **(C)** time variation of similarity of form of coupling functions ρ(*t*), **(D)** coupling functions *q*_2_(ϕ_*r*_, ϕ_*h*_) at specific time points, indicated by the gray arrows, that correspond to maximal and minimal frequency of respiration, and **(E)** the mean, i.e., time-averaged coupling functions for all three breathing patterns under investigation.

In Figures 6A–D we present the results for the cardiorespiratory interaction when the respiration varies according to sine law. In Figure 6A we give the wavelet transform of the respiration for comparison. In Figure 6B the time-variation of the coupling strength from the first oscillator (the respiratory system) to the second one (cardiac system) is presented. We can see here that the coupling strength has a minimum where the frequency of respiration is maximal and a maximum where the frequency of the respiration is minimal, i.e., the time-variability of the coupling strength resembles an inverse of the sine wave respiration. This confirms known results that the cardiorespiratory coupling strength is higher on slower breathing (Stankovski et al., 2012, 2013). In Figure 6C we present the time-variation of the index for similarity of form of coupling functions, which again follows the inverse of the sine wave respiration. This demonstrates that the form of the coupling function, and thus the underlaying cardiorespiratory mechanism, is time-varying and is following the deterministic perturbation we induced on the respiration. Again the higher similarity is associated with lower respiration frequencies and slower breathing. In Figure 6D we give the coupling functions at specific time points that correspond to maximal and minimal frequencies of respiration. Here we can also follow the time evolution of the coupling function close to the minimal frequency of respiration. The qualitative 3D representation of the coupling functions in Figure 6D shows visually consistent values of the coupling strength amplitude and similarity of the form of the functions as compared to the quantitative values presented in Figures 6B,C.

Finally, in Figure 6E we present the form of the time-averaged coupling function for all three breathing patterns under investigation. By comparison, we see that the form of the three functions is qualitatively similar, with larger deviations for the a-periodic breathing in comparison to the free and sine breathing. From Figure 6 we can see that the reconstructed cardiorespiratory coupling functions are described by complex functions whose form changes quantitatively over time and with the change of frequency of respiration. This implies that the interactions of the cardiorespiratory system can themselves be time-varying processes. In particular, the form of the coupling function indicates that when it is high for the respiration phase between 3π/2 and π/2 (Figure 6E), then the respiration accelerates the cardiac oscillations. Similarly, when the coupling function is low for respiration phase between π/2 and 3π/2 (Figure 6E), then the respiration decelerates the cardiac oscillations. These inferred coupling functions describe in detail the cardiorespiratory interaction mechanism.

## 4. Discussion and Conclusion

In this study we have tackled the longstanding problem of choosing the right size of time window when analyzing dynamical time-series. We proposed new methodology for determination of the time window and the propagation parameter within the framework of the Dynamical Bayesian Inference method. We tested the method first on the case of coupled phase oscillators and then for the case of coupled limit-cycle oscillators. We then applied the methodology on cardiorespiratory interaction for three cases of respiration—free breathing, controlled breathing following sine law and controlled breathing following an a-periodic time-variation. We obtained the coupling functions and confirmed their complex form that changes quantitatively over time.

To some extent the problem of time window determination is an ill-posed question, especially in experimental analysis, because in theory it is very hard to find a general solution. There can be very different systems, with very different types of time-variabilities acting on different parts of the systems. Nevertheless, the reality is that often there is time-variability and one needs not to ignore, but to do something about it. For this reasons, the solution we proposed is modeled and scaled to an important, albeit specific and not general, problem of cardiorespiratory interaction. In particular we took the systems to be oscillatory, hence we used the phase dynamics representation, and we assumed that the time-variation are slowly changing in respect to the oscillating frequencies. This allowed us to model a dynamic situation often encountered in the cardiorespiratory interaction. Additionally, by using second order Fourier expansion for the model base functions, we encountered limitations in inferring highly non-linear dynamics and very slow trends.

On the analysis of a predefined interacting phase oscillators we developed detailed conditions for the time window determination. These could not be determined exactly in an unknown system of coupled limit-cycle oscillators (like the example of the Poincaré oscillators in section 2.5), however, based on the phase oscillator acting as a limiting model, the analysis showed that one can find the boundaries and inequalities from which the time window can be determine in these cases. The use of the inferred covariance matrix as an indicator of the goodness of fit may become too strict and imprecise if there are large variations arising from the noise. In such case one should apply other stochastic methods in combination with this method to determine the effect of the noise and to ascertain the role of the covariance for determination of the time window. When dealing with biological open oscillatory systems, one might encounter a case where there is time-variability of the time-variability. In such case, the presented methodology may be applied recursively, for the different levels of time-variability observed.

The application to the cardiorespiratory interaction lead to some novel results, some were extended, and some results were consistent with previous findings. Namely the change of the coupling strength with slower breathing is known, and now we extended this to show that this variations appear continuously and were following the sine perturbation. A new insight is that the index for similarity of cardiorespiratory coupling functions is also higher with slower breathing and was following continuously the sine perturbation. In fact, in this analysis set up of the cardiorespiratory interaction, it was found that both the coupling strength and the similarity index were changing similarly, and in accordance with the perturbation (which is not the case in general). The inferred form of the cardiorespiratory coupling function, in the three types of breathing observed, was found to be consistent with what has been observed in previous studies. Interestingly, even though the window length determined for the three different types of breathing was quite different in length (free *t*_*w,opt*_ = 9*s*, sine *t*_*w,opt*_ = 62*s* and a-periodic *t*_*w,opt*_ = 15.6*s*), the form of the coupling functions (Figure 6E) were qualitatively very similar.

During slower breathing, the form of the coupling functions changes predominantly along the respiration phase axis and is relatively constant along cardiac phase axis. The latter suggests that this coupling is predominately determined by the direct influence of respiration on the heart. This is most visible on the coupling function during low frequency parts of the breathing following sine law (Figure 6D) and not so visible for the aperiodic breathing which is at higher respiration frequencies. In physiology, this influence of the respiration frequency to the variability of the heart rate has been attributed to Respiratory Sinus Arrythmia (RSA) (Hirsch and Bishop, 1981), and various studies have linked the cardio-respiratory coupling with RSA (Iatsenko et al., 2013; Schulz et al., 2013; Kralemann et al., 2013a). Recent physiological studies discussed that the main function of the RSA is to improve cardiac efficiency while maintaining physiological levels of arterial CO_2_ (Elstad et al., 2018). Our findings confirm these previous findings that RSA is more pronounced during slow deep breathing (Hirsch and Bishop, 1981).

Needles to say, even though this study was presented for oscillatory interactions and in particular for cardiorespiratory interaction, its implications span much widely. Some of the solutions proposed with this methodology for time window determination are relevant and can be used for other oscillatory interactions, for other methods of time-series analysis, and for other dynamical systems with time-variability, more generally.

## Data Availability Statement

The datasets generated for this study are available on request to the corresponding author.

## Ethics Statement

The studies involving human participants were reviewed and approved by Ethical committee, Faculty of Medicine, Saints Cyril and Methodius, Skopje, Macedonia. The patients/participants provided their written informed consent to participate in this study.

## Author Contributions

DL and TS conceived and planned the research and wrote the first draft of the manuscript. DL designed the methodological procedure and completed the analysis with advice and supervision from TS, HS, and MG. All authors discussed the results and contributed to the editing of the manuscript.

## Conflict of Interest

The authors declare that the research was conducted in the absence of any commercial or financial relationships that could be construed as a potential conflict of interest.
